# Two New Species of the Genus *Meleonoma* (Lepidoptera, Autostichidae) from China, Revealed by Morphological and Phylogenetic Evidence †

**DOI:** 10.3390/insects17060649

**Published:** 2026-06-19

**Authors:** Xiaoju Zhu, Xiuxiu Zhu, Shuxia Wang

**Affiliations:** 1 College of Plant Protection, Shandong Agricultural University, Tai’an 271018, China; xjzhu@sdau.edu.cn; 2 College of Life Sciences, Nankai University, 94 Weijin Road, Tianjin 300071, China; xiuxiuz@163.com

**Keywords:** Periacminae, classification, new species, mitochondrial genome, phylogenetic analysis

## Abstract

*Meleonoma* belonging to the subfamily Periacminae is one of the most species-rich genus in the family Autostichidae. In this study, we described two new species: *M. latizona* sp. nov. and *M. serrulata* sp. nov. A phylogenetic analysis of these and closely related species was conducted based on mitochondrial genes to achieve a better understanding of their taxonomic status of the two new species.

## Simple Summary

Two new species belonging to the genus *Meleonoma* were identified in China. Detailed illustrations of adults, the male and female genitalia, as well as the complete mitochondrial genome maps of both taxa are provided.

## 1. Introduction

The genus *Meleonoma* Meyrick was established by Meyrick under the family Oecophoridae [1], with *Cryptolechia stomata* as the type species [2]. Clarke [3] transferred it to Cosmopterigidae, a placement subsequently adopted by several researchers [4,5,6]. Lvovsky [7] described the new genus *Acryptolechia* in the family Cryptolechiidae, with *Cryptolechia malacobyrsa* [8] as its type species. Heikkila et al. [9] treated Cryptolechiinae as a subfamily within the family Depressariidae, although *Meleonoma* was not included in their study. Later, Lvovsky [10] synonymized *Acryptolechia* with *Meleonoma* and assigned it to the family Lypusidae, a placement that was subsequently followed by Park & Park [11]. Due to this unstable systematic placement, Yin & Wang [12,13] treated *Meleonoma* as a genus in the family Oecophoridae within Gelechioidea, following its original treatment. Kitajima & Sakamaki [14,15] placed *Meleonoma* in the family Oecophoridae in their study of the genus in Japan. Wang & Li [16] reconstructed the molecular phylogeny of Gelechioidea based on one mitochondrial marker (COI) and seven nuclear markers. Their analyses recovered that *Meleonoma* [1] and *Phaulolechia* [17] formed the tribe Meleonomini, while *Periacma* [18], *Irepacma* Moriuti et al. [19], and *Epiracma* [20] constituted the tribe Periacmini. Both tribes belong to the subfamily Periacminae under the family Autostichidae, which is substantiated by recent studies [21,22,23].

The genus *Meleonoma* is characterized by the narrow to broad lanceolate forewings always yellow or black; the gnathos present or absent, if present, being a circle (in some species with a ventral plate), or either separated, or indistinct ventrally, the sacculus, in most species, separated from the valva entirely or distally; ductus bursae membranous, partly or entirely sclerotized, corpus bursae with or without granules, signum present or absent. A total of 217 valid species of *Meleonoma* have been described worldwide to date, most of which are distributed in the Palearctic and Oriental regions [24], and these taxa can be assigned to one of nine species groups [24,25].

Although numerous new species of the genus *Meleonoma* have been continuously reported in recent years [21,22,23,25,26], taxonomic research on this genus still largely depends on traditional morphological characters. Morphological data alone are sometimes insufficient to address challenges in accurate species delimitation and cryptic species recognition, particularly for resolving species-group boundaries [27], due to confounding factors such as intraspecific variation and high interspecific morphological similarity. Therefore, it is highly necessary to supplement morphological identification with molecular evidence. Despite this rich species diversity, molecular resources for *Meleonoma* remain extremely limited—only one complete mitochondrial genome has been publicly released to date, which to a certain extent hinders in-depth systematic research, such as elucidating phylogenetic relationships. The present study aims to explore more new species of the genus *Meleonoma*, while also filling the research gap of mitochondrial genome; the mitochondrial genome data for *Meleonoma* and its allied genera were provided to supplement morphological identification.

## 2. Materials and Methods

### 2.1. Sample Collection

A total of 35 specimens were collected by light traps using 250 W high-pressure mercury lamps (Yaming, Shanghai, China) on a white sheet in the mountainous areas of China (Henan, Hubei, Sichuan), with permission from local government officials. Ten specimens of the genus *Meleonoma*, one specimen of the genus *Ripeacma*, and one specimen of genus *Periacma* were preserved in absolute ethanol and stored at −20 °C for DNA extraction (Table S1). The type series of the new species are deposited at the Insect Collection of Tianjin Natural History Museum (TJNHM), Tianjin, China.

### 2.2. Morphological Analyses

Morphological terminology in the descriptions follows Wang et al. [24]. Genitalia dissection and mounting follow the methods introduced by Li [28], stained using Eosin Y (Solarbio, Beijing, China). Images of adults were taken with a Leica M205A stereomicroscope and genitalia were prepared using a Leica DM750 microscope, equipped with Leica Application Suite 4.2 software (Leica, Wetzlar, Germany). All images were processed with Photoshop CC (Adobe, San Jose, CA, USA).

### 2.3. DNA Extraction and Sequencing

Ten *Meleonoma* individuals, one *Periacma* individual, and one *Ripeacma* individual were sampled for mitogenome sequencing (Table S1). The Genomic DNA was extracted from legs or partial body of dried specimens using the QIAGEN^®^DNeasy Blood & Tissue Kit (Qiagen, Hilden, Germany). The genome sequencing was performed by Shanghai Personal Biotechnology Co., Ltd. (Shanghai, China), on Illumina NovaSeq X plus platform (Illumina, Inc., San Diego, CA, USA), using 150 bp paired-end reads. We used fastp [29] to remove low-quality reads.

### 2.4. Mitogenome Assembly and Annotation

The sequences of mitogenome were assembled using mitoZ 2.3 [30] with default settings. Transfer RNA (tRNA) genes were annotated using MITOS2 webserver (available at http://mitos.bioinf.uni-leipzig.de/index.py, accessed on 12 January 2026) with an invertebrate mitochondrial genetic code. Protein-coding genes (PCGs) and ribosomal RNA (rRNA) were annotated by alignment with the homologous genes of previously published mitochondrial genomes of *Meleonoma* mirabilis in GenBank (accession numbers: NC_058014). Then, mitogenome maps were depicted using Proksee [31] (available at https://proksee.ca/, accessed on 12 January 2026).

### 2.5. Sequence Analyses

We used MEGA X [32] to calculate the codon usage of 13 PCGs and the nucleotide compositions of the whole mitogenome. The bias of the nucleotide composition for each mitogenome was measured by AT-skew [(A − T)/(A + T)] and GC-skew [(G − C)/(G + C)]. The non-synonymous substitution rate (Ka) and synonymous substitution rate (Ks) of each PCG was calculated using DnaSP 6.12.03 [33], and the ratio of Ka/Ks was used to represent the evolution rate of each PCG.

### 2.6. Genetic Distances Estimation and Phylogenetic Reconstruction

To perform the different analyses, three datasets were obtained: (A) a dataset of the COI gene, including nineteen *Meleonoma* species (in addition to the ten species mentioned above, nine *Meleonoma* species were also downloaded from NCBI); (B) same as dataset A but with ten more species form its allied genera under the subfamily Periacminae, downloaded from NCBI, and *Apethistis uncinata* as an outgroup; (C) a dataset combining thirteen PCGs (ATP6, ATP8, COI, COII, COIII, CYTB, ND1, ND2, ND3, ND4, ND4l, ND5 and ND6) of the mitogenome, in addition to the twelve species mentioned above obtained in the current study, with *M. mirabilis* (*Meleonoma*), *R. umbellata* (*Ripeacma*) and *P. orthiodes* (*Periacma*) included.

Genetic distances were calculated under the Kimura 2-parameter model [34] using MEGA X. Dataset A was used to calculate the intrageneric genetic distances, and the datasets B and C were analyzed by maximum likelihood (ML) and Bayesian inference (BI). For ML analyses, 1000 ultrafast bootstraps were performed using IQ-TREE 3.0.1 [35] under the best-fit partition models (Table S2). For BI tree construction, the best-fit partitioning scheme and nucleotide substitution models were calculated using PartitionFinder 2.1.1 [36], and Bayesian inference was performed using MrBayes 3.2.7a [37].

## 3. Results

Taxonomy

Class Insecta Linnaeus, 1758.

Order Lepidoptera Linnaeus, 1758.

Superfamily Gelechioidea Stainton, 1854.

Family Autostichidae Le Marchand, 1947.

Genus *Meleonoma* Meyrick, 1914.

Type species: *Cryptolechia stomota* Meyrick, 1910.

= *Acryptolechia* Lvovsky, 2010, syn.

Type species: *Cryptolechia malacobyrsa* Meyrick, 1921.

### 3.1. Morphological Results

#### 3.1.1. *Meleonoma latizona* Zhu & Wang, sp. nov.

Zoobank: urn:lsid:zoobank.org:act:FC71B374-0613-43D4-87BF-B00AFA42E8F6

Figure 1

Type material. CHINA, Sichuan: Holotype ♂, Jinding, Mt. Fanjing, 2200 m, 30.V.2002, leg. XP Wang, slide No. W01265. Paratypes: Sichuan: 1♂2♀, Huguosi, Mt. Fanjing, 1390 m, 28.V.2002, leg. XP Wang, slide Nos. YAH15463♂, YAH15462♀, W05106♀; Henan: 1♂1♀, Baotianman, Neixiang County, 1200 m, 30–31.V.2006, leg. X Zhang & JM Lv, slide Nos. ZXJ18187♀, ZXJ19185♂; 2♂1♀, Mt. Funiu, 1176 m, 2.VI.2024, leg. MJ Qi & YT Fu et al., slide No. ZXJ230039♂; Hubei: 1♂1♀, Shennongjia, 1800 m, 8.VII.2009, M Wang & Y Long, slide Nos. ZXJ18126♂, ZXJ19183♀.

Diagnosis. The new species is similar to *M. rostriformis* [38] in the forewing pattern and genitalia, and can be distinguished by the yellow longitudinal stripe extending from basal 2/5 of the costal margin being uninterrupted, whereas the yellow strip is interrupted in *M. rostriformis* [38]. It can also be distinguished from the latter by the costa of the costal part of the valva, with a large denticle in the male genitalia and a funnel-shaped antrum in the female genitalia; meanwhile, in *M. rostriformis* [38], the costa lacks a denticle and the antrum is inverted U-shaped.

**Figure 1:**
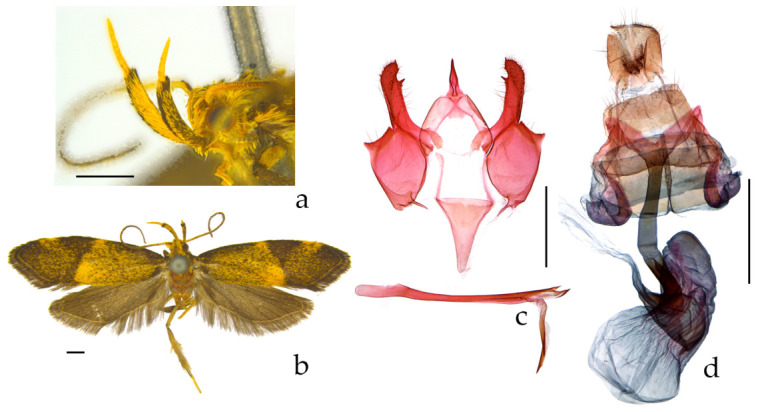
*M. latizona* sp. nov.: (**a**) head; (**b**) dorsal surface of adult; (**c**) male genitalia, slide No. ZXJ19185, paratype; (**d**) female genitalia, slide No. ZXJ19183, paratype. Scales (**a**–**d**) = 1.0 mm.

Description. Adult (Figure 1a,b). Forewing length 18.0–21.0 mm.

Head yellow, dark brown medially on vertex and occiput. Labial palpus yellow; first segment with dark brown scales on outer and dorsal surfaces; second segment with dark brown scales on outer and dorsal surfaces, dark brown at apex; third segment approximately half length of second segment. Antenna yellow; scape with scattered dark brown scales on dorsal surface; flagellum annulated with dark brown on dorsal surface.

Mesonotum dark brown, yellow marginally; tegula dark brown, yellow apically. Legs yellow, with exceptions on ventral surface: coxa of foreleg black medially, femur and tibia black except tibia yellow marginally, tarsus black at middle of basal tarsomere and at apical three tarsomeres, femora of mid- and hindlegs mixed with black scales, tibia of midleg black except yellow at middle and at apex, tibia of hindleg black mixed with yellow scales, tarsus of midleg black at base of basal tarsomere and at base of apical one tarsomere, tarsus of hindleg mixed sparse black scales. Forewing elongate lanceolate, apex narrowly rounded; ground colour dark brown, overlaid with yellow maculations; costal margin with a diffused yellow longitudinal stripe extending from basal 2/5 obliquely downwards to end of fold, gradually widened towards the wing base; distal portion with a broad blackish brown transverse band, the central part of which is interspersed with yellow scales; the upper margin of the band extends from the middle of costal margin to the wing apex, interrupted medially by a well-defined yellow spot dotted with sparse dark brown scales, while the lower margin extends from the apex of the fold along the outer margin to the wing apex; cell with a dark brown spot near middle; dorsum with a dark brown spot at the wing base; fringe dark brown, mixed with yellow scales basally. Hindwing and fringe greyish brown.

Male genitalia (Figure 1c). Uncus flame-shaped, pointed at apex. Tegumen widened medially, with a V-shaped anterior emargination, blunt at apex. Costal part of valva uniformly in width except slightly narrower apex, setose ventrodistally, ventral margin fused with sacculus in basal 1/3, with a row of different denticles from before distal 1/3 to apex, ventral surface with small denticles extending from basal 1/3 to before distal 1/3, then spreading upwards to before apex; costa with a row of small denticles form basal 2/5 to distal 1/3, distal 1/3 with a large denticle, then deeply concave from beyond distal 1/3 to before apex, apex with a denticle; transtilla banded, connected medially by membrane. Sacculus trapezoidal, concave and serrated on outer margin, apex with a process dorsally and ventrally respectively. Saccus triangular, rounded at apex, nearly twice length of uncus. Juxta narrowly linked basally, lateral arm slender, pointed at apex. Aedeagus slender, about twice length of costal part of valva, with basal 1/4 wider than distal 3/4, concave before apex; forming two spines at apex; cornuti placed in vesica, being two spines, different in size.

Female genitalia (Figure 1d). Papillae anales sub-quadrate, setose. Apophyses posteriores approx. 3.5 times as long as apophyses anteriores. Eighth sternal plate rectangular, marginally with long setae on posterior margin. Lamella antevaginalis sub-oval. Antrum funnel-shaped. Ductus bursae sclerotized, nearly uniform in width, with a sclerite extending from near entrance of corpus bursae to posterior margin of corpus bursae. Corpus bursae irregular, with fine folds; ductus seminalis arising from corpus bursae; signum absent.

Distribution. China (Henan, Hubei, Sichuan).

Etymology. The specific epithet is derived from the Latin *latizonus*, referring to the distal part of the forewing with a wide dark brown band.

Note. The new species can be classified into the *segregnatha* group.

#### 3.1.2. *Meleonoma serrulata* Zhu & Wang, sp. nov.

Zoobank: urn:lsid:zoobank.org:act:90E5D564-571E-42C5-9CFE-BE96F5665321

Figure 2

Type material. CHINA, Sichuan: Holotype ♂, Songpinggou, Mao County, 4.VII.2021, leg. S Yu et al., slide No. ZXJ20009. Paratypes: 5♂6♀, 4−6. VII.2021, other same data as holotype, slide No. ZXJ20003♂, ZXJ20180♀, ZXJ20181♂, ZXJ20182♀, ZXJ23068♂, ZXJ23071♀.

Diagnosis. The new species is similar to *M. dorsoprojecta* [38] in the forewing pattern, and can be distinguished in the male genitalia by the flame-shaped uncus pointed at the apex, the sacculus lacking a dorsal process, and the aedeagus being longer than the costal part of the valva; in the female genitalia, the corpus bursae lacks a signum. In *M. dorsoprojecta* [38], the clavate uncus is rounded at the apex, the sacculus has a dorsal process, and the aedeagus is shorter than the costal part of the valva; the corpus bursae has two signa.

**Figure 2:**
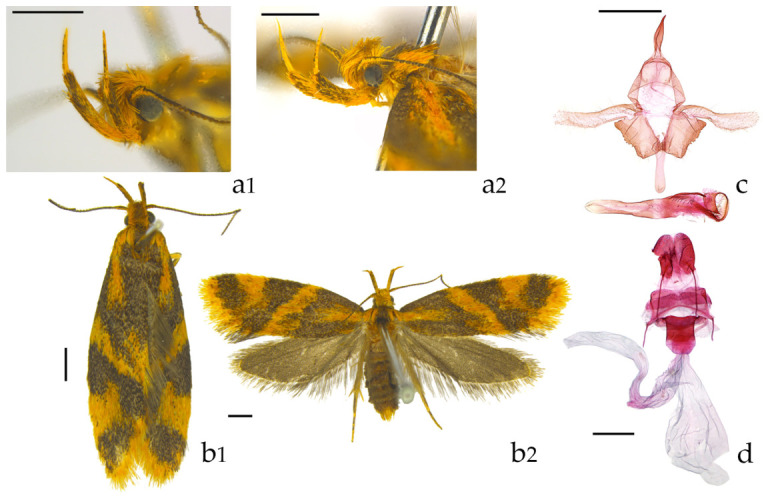
*M. serrulata* sp. nov. ((**a1**), **b1**♂, (**a2**), **b2**♀): (**a1**,**a2**) head; (**b1**,**b2**): dorsal surface of adult; (**c**) male genitalia, slide No. ZXJ20009, holotype; (**d**) female genitalia, slide No. ZXJ20182, paratype. Scales (**a1**,**a2**,**b1**,**b2**) = 1.0 mm; Scales (**c**,**d**) = 0.5 mm.

Description. Adult (Figure 2(a1,a2,b1,b2)). Forewing length 14.0–16.0 mm.

Head orange yellow, tipped with greyish brown on occiput. Labial palpus orange yellow; second segment mixed with dark brown scales in distal 2/3; third segment about half length of second segment, mixed with dark brown scales in distal 2/3. Antenna orange yellow; scape dark brown on dorsal surface; flagellum with dorsal surface dark brown, ventral surface orange yellow alternated with black on ventral surface except orange yellow basally.

Mesonotum dark brown, orange yellow marginally; tegula orange yellow. Legs yellow, with exceptions on ventral surface: coxa of foreleg mixed with dark brown scales, femur and tibia dark brown, tarsus yellow except dark brown at middle of basal tarsomere and dark brown at apical three tarsomeres, midleg with femur and tibia dark brown, except yellow at apex of femur and at base of basal tarsomere, as well at basal two tarsomeres and apical one tarsomere, hindleg with femur and tibia dark brown except yellow at base of basal tarsomere and at apices of basal two tarsomeres and yellow at apical two tarsomeres. Forewing lanceolate, apex rounded; ground color orange yellow, with black scales, dense black scales forming a black stripe along basal 1/3 of costal margin, then extending downwards to crossing cell above fold; median fascia black, extending obliquely from before middle of costal margin downwards to tornus, narrower in its middle; a black patch placed at apex; dorsal margin with a black stripe extending from base to before end of fold; fringe orange yellow, black at tornus. Hindwing and fringe grey brown.

Male genitalia (Figure 2c). Uncus flame-shaped, pointed at apex. Gnathos sclerotized laterally, membranous anteriorly. Tegumen widest at middle, narrowed laterally. Costal part of valva nearly equal in width, setose; costal band narrowed distally to reaching before apex; transtilla triangular, pointed at apex. Sacculus trapezoidal, serrated on apical margin. Saccus tubular, longer than uncus. Juxta U-shaped, with lateral arm triangular. Aedeagus tubular, longer than costal part of valva, with fine wrinkles dorsally at distal 2/5, forming a spine dorsally in distal 1/5; a curved sclerotized band at preapex, pointed at apex; cornutus absent.

Female genitalia (Figure 2d). Papilla analis sub-rectangular. Apophysis posterioris approx. 2.0 times as long as apophysis anterioris. Eighth tergite sub-rectangular, slightly concave medially on posterior margin. Eighth sternal plate with a triangular median groove, lined with long stout setae on posterior margin. Lamella antevaginalis band-shaped. Ostium bursae concave medially. Antrum cup-shaped, longer than ductus bursae. Ductus bursae short, membranous, straight. Corpus bursae pyriform, membranous; signum absent.

Distribution. China (Sichuan).

Etymology. The specific epithet is derived from the Latin *serrulatus*, referring to the sacculus serrated on apical margin.

Note. The new species can be classified into the *malacobyrsa* group.

### 3.2. Molecular Results

#### 3.2.1. Mitogenome Features of the *Meleonoma* Species

**Figure 3:**
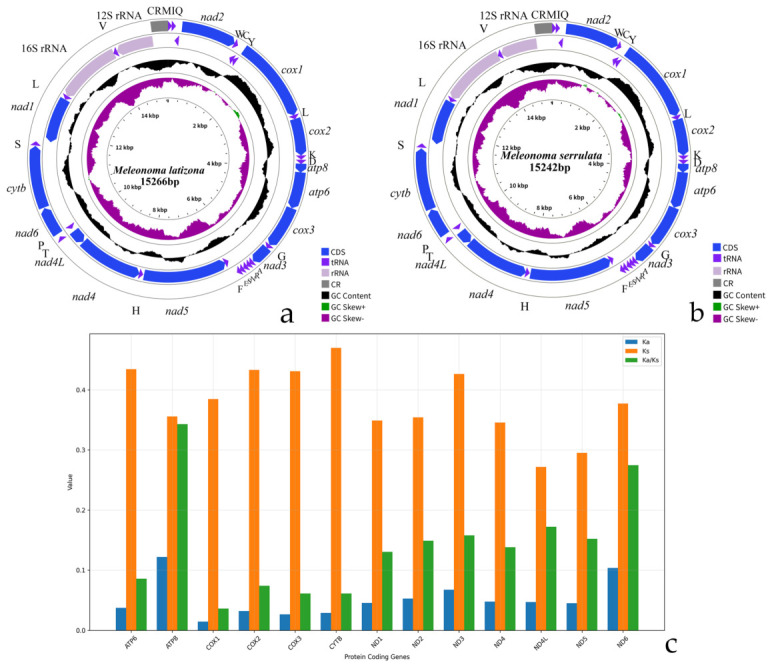
Complete mitogenome maps. (**a**) *M. latizona* sp. nov.; (**b**) *M. serrulata* sp. nov. The direction of gene transcription is indicated by the arrows on the strands. PCGs and rRNAs are represented by normative abbreviations, while tRNAs are indicated by single-letter abbreviations. (**c**) Evolution rate of each PCG of the 11 *Meleonoma* species.

The entire length of the ten mitogenomes representing the ten *Meleonoma* species ranged from 15,197 bp to 15,355 bp (Table 1). Each mitogenome contained 37 typical insect mitochondrial genes: 22 tRNAs, 13 PCGs, 2 rRNAs, and 1 A + T-rich region (Figure 3). Among these genes, 23 are located on the majority strand (J-strand), whereas the remaining 14 genes are encoded on the minority strand (N-strand). All mitogenome sequences exhibited a significant A + T nucleotide bias. The overall A + T content of the ten *Meleonoma* species ranged from 77.22% to 80.31%, with the highest value observed in *M. latizona* sp. nov. (80.31%) and the lowest in *M. microbyrsa* (77.22%) (Table 1). For base composition skewness, all AT-skew values were negative, ranging from −0.0535 to −0.0246, indicating a higher proportion of T than A in the majority strand. Meanwhile, all GC-skew values were also negative, ranging from −0.2927 to −0.2185, reflecting a higher content of C relative to G in the mitogenomes (Table 1).

PCGs of the ten *Meleonoma* species were mainly initiated with ATN (ATA, ATT, ATG, and ATC), except for COI (CGA). Notably, a rare GTT initiation codon was observed in the ND1 gene of three species—*M. deflecta*, *M. torophanes*, and *M. facialis*—a phenomenon rarely documented in insect mitochondrial genomes, which constitutes a distinctive variation within the genus *Meleonoma*. The termination codons were TAA in most species. In particular, the ND1 gene of *M. torophanes*, *M. facialis*, and *M. serrulata* sp. nov. used TAG as its termination codon, and ND3 gene of *M. deflecta* used TAG as its termination codon. The same pattern was found in the ND4l gene of *M. deflecta*, *M. torophanes*, and *M. microbyrsa*. For COX1, COX2, ND4, and ND5, a single T residue served as an incomplete termination signal, a well-known common feature of lepidopteran mitogenomes that facilitates efficient genome compaction (Table S3). The value of Ka/Ks for each PCG was less than 0.35; ATP8 exhibited the largest value of Ka/Ks, and COI had the lowest value of Ka/Ks (Figure 3).

The amino acids’ frequency and relative synonymous codon usage (RSCU) values for PCGs in the ten mitogenomes are summarized in Figure S1. The most common amino acid in the ten mitogenomes is Leu2, while the least frequently used amino acid is Ser1. In the mitogenome of *M. serrulata* sp. nov., 24 codons exhibit positive codon usage bias (RSCU > 1), indicating that these codons are used more frequently, 36 codons show a negative codon usage bias (RSCU < 1). In *M. latizona* sp. nov., 27 codons exhibit positive codon usage bias (RSCU > 1), indicating that these codons are used more frequently, while 33 codons show a negative codon usage bias (RSCU < 1). Among these, two codons, UCG (Ser2) and Ser1 (Arg), are not used only in the *M. serrulata* sp. nov. mitogenome, and CUG (Leu1) and CCG (Pro) are not used in the *M. latizona* sp. nov. mitogenome.

#### 3.2.2. Genetic Distances and Phylogenetic Relationships

**Figure 4:**
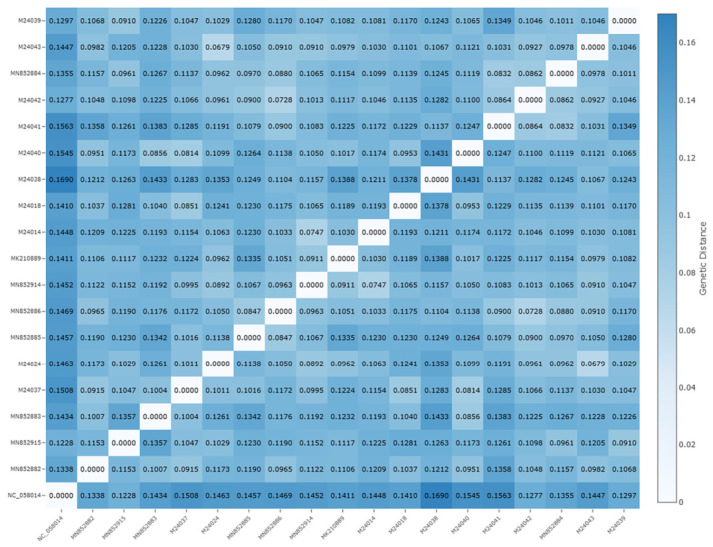
Heatmap of Kimura 2-parameter (K2P) genetic distances among the mitochondrial COI sequences of the *Meleonoma* species.

**Figure 5:**
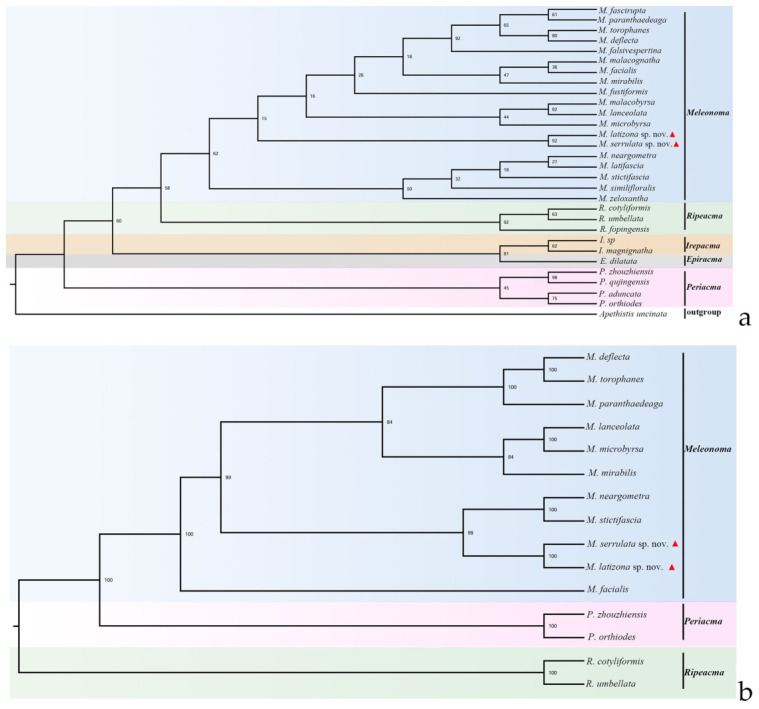
Maximum likelihood (ML) and Bayesian inference (BI) phylogenetic tree of *Meleonoma* based on COI datasets (**a**) and on mitogenomes (PCG datasets) (**b**). Numbers near branches refer to bootstrap support values and Bayesian posterior probabilities.

The genetic distance among 19 species was analyzed based on dataset A with 666 bp aligned fragments. The results (Figure 4) indicate that all included species possess highly species-specific DNA barcodes. The interspecific genetic distance within the genus *Meleonoma* is 0.067887~0.168986. The minimum genetic distances based on the COI gene between *M. latizona* sp. nov. and its congeners, as well as between *M. serrulata* sp. nov. and its congeners, were both 0.0679. Additionally, their interspecific genetic distances are higher than the 3% threshold for Lepidoptera [39], confirming the validity of the species at the molecular level. The phylogenetic relationship of the *Meleonoma* species and its allied genus were reconstructed based on datasets B and C. Our phylogenetic analyses strongly supported the monophyly of the genus *Meleonoma*, and identified the two new species within this clade, providing conclusive molecular evidence that the two new species are correctly placed within *Meleonoma* (Figure 5).

## 4. Discussion

The forewing morphological characteristics of the new species *M. serrulata* sp. nov. are commonly present in both *Meleonoma* and *Periacma* (Autostichidae, Periacminae) [24,40]. The two genera can be distinguished by the number of labial palp segments in male adults: two segments in *Periacma* and three segments in *Meleonoma*. However, females of both genera consistently possess three-segmented labial palps and exhibit highly similar morphological features [41]. Therefore, accurate species identification cannot be reliably achieved relying solely on traditional morphological methods, which further emphasizes the necessity and urgency of integrated taxonomic research.

Both new species can be confidently assigned to the genus *Meleonoma* based on shared morphological characteristics: three-segmented labial palps in males, lanceolate forewings, a reduced gnathos, and the absence of a dorso-proximal process on the costal part of the valva in male genitalia. In contrast, species of *Periacma* are characterized by a gnathos with a well-developed ventral plate and a distinct dorso-proximal process on the costal part of the valva. Molecular phylogenetic evidence further supports the generic placement of both new species within *Meleonoma* (Figure 5).

To validate the specific status of the two new species and confirm their placement within the subfamily Periacminae, we performed phylogenetic analyses based on COI barcodes and complete mitochondrial genomes. The results strongly supported that both taxa represent valid new species belonging to the genus *Meleonoma*. Our molecular phylogenetic results are largely consistent with the phylogenetic framework proposed by Wang & Li (2020) [16]. The monophyly of *Meleonoma* is stably recovered across all analyses (Figure 5). In the present study, the 19 sampled *Meleonoma* species form a well-supported monophyletic clade in the COI tree, clearly separated from the related genera *Ripeacma*, *Irepacma* and *Periacma*. A direct comparison between the COI and 13 PCG datasets demonstrates the superior performance of mitogenomic data in resolving phylogenetic relationships within *Meleonoma* with 100% bootstrap support.

To date, most described *Meleonoma* species have been divided into nine species groups based on morphological and genitalic characters: the *M. malacobyrsa* group [24,26,42], *M. facialis* group [22,24,27,42], *M. annulignatha* group [24,27,43], *M. segregnatha* group [21,24,27,43], *M. dentivalvata* group [23,24,44], *M. fasciptera* group [24,27,44], *M. jigongshanica* group [24,25], *M. acutiuscula* group [24,25,27], and *M. puncticulata* group [25,27]. Among them, *M. serrulata* sp. nov. is assigned to the *M. malacobyrsa* group [24,26,42] based on its diagnostic traits, including a lanceolate forewing with an orange-yellow ground color and a broad black median fascia. *M. latizona* sp. nov. is placed in the *M. segregnatha* group [21,24,27,44] according to its dark brown forewing overlain with yellow maculations. The assignment of the two species to their respective species groups is currently based on their diagnostic morphological traits. However, in the phylogenetic tree (Figure 5), these two species cluster together and *M. serrulata* sp. nov. is not resolved into the *M. malacobyrsa* group. The same issue is also observed in the *dentivalvata* and *fasciptera* species groups (Figure 5a). We hypothesize that this is because the COI gene contains limited genetic information and is insufficient for resolving species groups within the genus *Meleonoma*, necessitating the incorporation of additional genetic data. In contrast, the mitochondrial genome (Figure 5b), which harbors substantially more genetic information, successfully delimited the *dentivalvata* and *fasciptera* groups. Nevertheless, *M. latizona* sp. nov. and *M. serrulata* sp. nov. still formed a well-supported clade, and *M. serrulata* sp. nov. did not cluster with the *malacobyrsa* group. This suggests that although the mitochondrial genome carries far more genetic information than the COI gene and can largely resolve species groups within *Meleonoma*, a robust and stable delimitation of species groups in this genus still requires the inclusion of more species groups and congeneric species. A comprehensive investigation into the intrageneric species group delimitation of *Meleonoma*, which will require adequate taxon sampling, is beyond the scope of the present study and will be addressed in our subsequent work.

## 5. Conclusions

Our study describes 2 new species of *Meleonoma* from China, increasing the total number of described species in this genus to 219. The integration of morphological and molecular data not only provides a more accurate species delimitation but also contributes to a better understanding of the taxonomy and systematics of *Meleonoma*. This work lays a foundation for future biodiversity surveys and phylogenetic relationships of Autostichidae in China.

## Figures and Tables

**Table 1:** Length and nucleotide composition of the whole mitogenomes of the ten *Meleonoma* species. Scales = 1.0 mm.

Species	Code	Length (bp)	T%	C%	A%	G%	A + T%	AT-Skew	GC-Skew
*M. lanceolata*	M24014	15,253	41.87	12.73	37.62	7.79	79.49	−0.0535	−0.2412
*M. deflecta*	M24018	15,320	40.97	13.39	38.05	7.59	79.02	−0.0369	−0.2763
*M. latizona* sp. nov.	M24024	15,266	41.97	12.14	38.34	7.55	80.31	−0.0452	−0.2331
*M. torophanes*	M24037	15,204	40.99	13.33	38.05	7.63	79.04	−0.0372	−0.2721
*M. microbyrsa*	M24038	15,315	39.56	14.72	37.66	8.06	77.22	−0.0246	−0.2927
*M. facialis*	M24039	15,197	41.36	13.13	37.74	7.78	79.09	−0.0457	−0.2558
*M. paranthaedeaga*	M24040	15,251	41.3	13.03	37.95	7.72	79.25	−0.0423	−0.256
*M. neargometra*	M24041	15,355	40.58	13.42	38.11	7.89	78.69	−0.0314	−0.2594
*M. stictifascia*	M24042	15,286	41.39	12.72	38.23	7.65	79.62	−0.0397	−0.2489
*M. serrulata* sp. nov.	M24043	15,242	41.74	12.21	38.22	7.83	79.96	−0.0440	−0.2185

## Data Availability

The original contributions presented in this study are included in the article/Supplementary Materials, and the GenBank accession numbers of the newly sequenced species are listed in Table S1. Further inquiries can be directed to the corresponding authors.

## Supplementary Materials

The following supporting information can be downloaded at: https://www.mdpi.com/article/10.3390/insects17060649/s1. Figure S1: Patterns of codon usage in the mitogenomes of the ten *Meleonoma* species. The X-axis shows the codon families and the Y-axis shows the total codons. Table S1: Details of samples used in this study; Table S2: The best model for each partition of Datasets A and B; Table S3: Start and termination codons of PCGs in the mitogenomes of ten *Meleonoma* species.

## Abbreviations

The following abbreviations are used in this manuscript:

TJNHM	Insect Collection of Tianjin Natural History Museum, Tianjin, China
RSCU	Relative synonymous codon usage
PCGs	Protein-coding genes
