# Supplementary material for: Two New Species of the Genus Meleonoma (Lepidoptera, Autostichidae) from China, Revealed by Morphological and Phylogenetic Evidence
Source: Insects. 2026 Jun 19;17(6):649. doi: 10.3390/insects17060649 (PMC13301608; doi:10.3390/insects17060649)
Supplement: Supplementary file 1 [file insects-17-00649-s001.zip › insects-4364800-supplementary.pdf]

Table S1. Details of samples used in this study.

Genus	Species	Code	Locality	Altitude	Collection date	Accession number (mt)	Accession number (COI)	Source
Meleonoma	*M. lanceolata*	M24014	Baotianman, Henan, China	414 m	14 Jul 2024	PZ534546	—	This study
	M. deflecta	M24018	Mt. Funiu, Henan, China	1236 m	22 Jun 2023	PZ534547	—	This study
	M. latizona sp. nov.	M24024	Mt. Funiu, Henan, China	1176 m	2 Jun 2024	PZ534556	—	This study
	M. torophanes	M24037	Baotianman, Henan, China	1359 m	25 Jun 2023	PZ534548	—	This study
	M. microbyrsa	M24038	Baotianman, Henan, China	609 m	13 Jul 2024	PZ534549	—	This study
	M. facialis	M24039	Baotianman, Henan, China	813 m	15 Jul 2024	PZ534550	—	This study
	M. paranthaedeaga	M24040	Baotianman, Henan, China	1279 m	23 Aug 2023	PZ534551	—	This study
	M. neargometra	M24041	Baotianman, Henan, China	1279 m	24 Jue 2023	PZ534552	—	This study
	M. stictifascia	M24042	Mt. Funiu, Henan, China	1176 m	21 Jun 2023	PZ534553	—	This study
	M. serrulata sp. nov.	M24043	Songpinggou, Mao County, Sichuan, China	2385 m	6 Jul 2023	PZ534554	—	This study
	M. mirabilis	—	China	—	—	NC_058014	—	GenBank
	M. falsivespertina	—	China	—	—	—	MN852882	GenBank
	M. malacognatha	—	China	—	—	—	MN852915	GenBank
	M. fascirupta	—	China	—	—	—	MN852883	GenBank
	M. similifloralis	—	China	—	—	—	MN852885	GenBank
	M. zeloxantha	—	China	—	—	—	MN852886	GenBank
	M. malacobyrsa	—	China	—	—	—	MN852914	GenBank
	M. fustiformis	—	Korea	—	—	—	MK210889	GenBank
	M. latifascia	—	China	—	—	—	MN852884	GenBank
Irepacma	*I. sp*	—	China	—	—	—	MN852907	GenBank
	I. magnignatha	—	China	—	—	—	MN852906	GenBank
Periacma	*P. zhouzhiensis*	P24017	Baotianman, Henan, China	813 m	25 Jun 2023	PZ534555	—	This study
	P. qujingensis	—	China	—	—	—	MN852925	GenBank
	P. aduncata	—	China	—	—	—	MN852924	GenBank
	P. orthiodes	—	China	—	—	MW697075	—	GenBank
Ripeacma	*R. cotyliformis*	R24019	Baotianman, Henan, China	1359 m	25 Jun 2023	PZ534557	—	This study
	R. umbellata	—	China	—	—	MN852933	—	GenBank
	R. fopingensis	—	China	—	—	—	MN852932	GenBank
Epiracma	*E. dilatata*	—	China	—	—	—	MN852895	GenBank

Genus	Species	Code	Locality	Altitude	Collection date	Accession number (mt)	Accession number (COI)	Source
Apethistis	*A. uncinata*	—	China	—	22 Mar 2016	—	MN852873	GenBank

Abbreviations: mt, mitochondrial genome; COI, mitochondrial cytochrome oxidase subunit I gene.

Table S2. The best model for each partition of Datasets B and C.

Datasets	Partition names	Best model
B	COI	GTR+F+I+G4
C	ATP6, ATP8, COI, COII, COIII, CYTB, ND1, ND2, ND3, ND4, ND4l, ND5, ND6	GTR+F+I+G4

Table S3. Start and termination codons of PCGs in the mitogenomes of *Meleonoma.*

Species	ATP6	ATP8	COX1	COX2	COX3	CYTB	ND1	ND2	ND3	ND4	ND4L	ND5	ND6
	**Start codon**												
M24014	ATG	ATC	CGA	ATG	ATG	ATG	ATT	ATT	ATT	ATG	ATG	ATT	ATT
M24018	ATG	ATT	CGA	ATG	ATG	ATG	GTT	ATT	ATT	ATG	ATG	ATT	ATT
M24037	ATG	ATT	CGA	ATG	ATG	ATG	GTT	ATT	ATC	ATG	ATG	ATT	ATT
M24038	ATG	ATT	CGA	ATG	ATG	ATG	ATT	ATT	ATT	ATG	ATG	ATT	ATC
M24039	ATG	ATT	CGA	ATG	ATG	ATG	GTT	ATC	ATT	ATG	ATG	ATT	ATC
M24040	ATG	ATA	CGA	ATG	ATG	ATG	ATG	ATT	ATC	ATG	ATG	ATT	ATT
M24041	ATG	ATC	CGA	ATG	ATG	ATG	ATT	ATT	ATT	ATG	ATG	ATT	ATC
M24042	ATG	ATC	CGA	ATG	ATG	ATG	ATT	ATT	ATC	ATG	ATG	ATT	ATT
M24043	ATG	ATT	CGA	ATG	ATG	ATG	ATT	ATT	ATC	ATG	ATG	ATT	ATA
M24024	ATG	ATC	CGA	ATG	ATG	ATG	ATT	ATT	ATT	ATG	ATG	ATT	ATA
	**Termination Codon**												
M24014	TAA	TAA	T	T	TAA	TAA	TAA	TAA	TAA	T	TAA	T	TAA
M24018	TAA	TAA	T	T	TAA	TAA	TAA	TAA	TAG	T	TAG	T	TAA
M24037	TAA	TAA	T	T	TAA	TAA	TAG	TAA	TAA	T	TAG	T	TAA
M24038	TAA	TAA	T	T	TAA	TAA	TAA	TAA	TAA	T	TAG	T	TAA
M24039	TAA	TAA	T	T	TAA	TAA	TAG	TAA	TAA	T	TAA	T	TAA
M24040	TAA	TAA	T	T	TAA	TAA	TAA	TAA	TAA	T	TAA	T	TAA
M24041	TAA	TAA	T	T	TAA	TAA	TAA	TAA	TAA	T	TAA	T	TAA
M24042	TAA	TAA	T	T	TAA	TAA	TAA	TAA	TAA	T	TAA	T	TAA
M24043	TAA	TAA	T	T	TAA	TAA	TAG	TAA	TAA	T	TAA	T	TAA
M24024	TAA	TAA	T	T	TAA	TAA	TAA	TAA	TAA	T	TAA	T	TAA

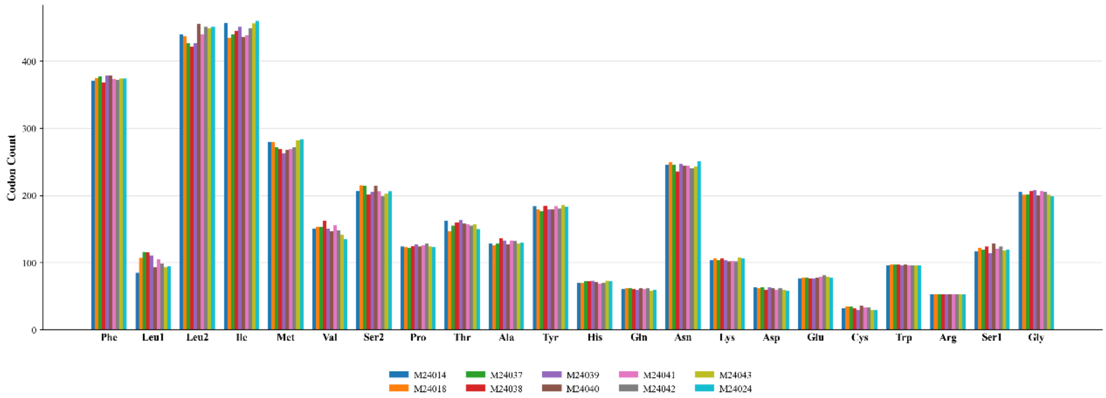

Figure S1. Patterns of codon usage in the mitogenomes of the *Meleonoma*. The X-axis shows the codon families and the Y-axis shows the total codons.
